# Donor–acceptor covalent organic frameworks for visible light induced free radical polymerization†
†Electronic supplementary information (ESI) available: Materials and methods, synthetic procedures, radical polymerization details, structure modelling details, Tables S1–S6, and Fig. S1–S17. See DOI: 10.1039/c9sc02601k


**DOI:** 10.1039/c9sc02601k

**Published:** 2019-08-05

**Authors:** Pradip Pachfule, Amitava Acharjya, Jérôme Roeser, Ramesh P. Sivasankaran, Meng-Yang Ye, Angelika Brückner, Johannes Schmidt, Arne Thomas

**Affiliations:** a Department of Chemistry/Functional Materials , Technische Universität Berlin , Hardenbergstraße 40 , 10623 Berlin , Germany . Email: pradip.pachfule@campus.tu-berlin.de ; Email: arne.thomas@tu-berlin.de; b Leibniz Institute for Catalysis , University of Rostock , Albert-Einstein-Str. 29a , 18059 Rostock , Germany

## Abstract

Covalent organic frameworks (COFs) are promising materials for applications in photocatalysis, due to their conjugated, porous and chemically stable architectures. Alternating electron donor–acceptor-type structures are known to enhance charge carrier transport mobility and stability in polymers and are therefore also interesting building units for COFs used as photocatalysts but also as photoinitiator. In this work, two donor–acceptor COFs using electron deficient 4,4′,4′′-(1,3,5-triazine-2,4,6-triyl)trianiline and electron rich thiophene-based thieno[3,2-*b*]thiophene-2,5-dicarbaldehyde or [2,2′-bithiophene]-5,5′-dicarbaldehyde linkers are presented. The resulting crystalline and porous COFs have been applied as photoinitiator for visible light induced free radical polymerization of methyl methacrylate (MMA) to poly-methyl methacrylate (PMMA). These results pave the way to the development of robust and heterogeneous systems for photochemistry that offers the transfer of radicals induced by visible light.

## Abstract 2

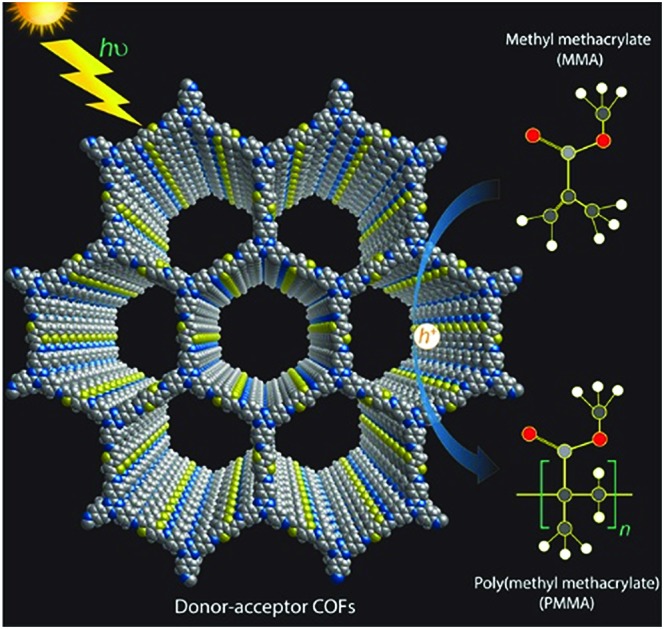
Crystalline and porous covalent organic frameworks (COFs) with donor-acceptor moieties in their backbone are utilized as initiators for visible light induced radical polymerization. The COFs are efficient photoinitiators, maintaining their structural integrity for several cycles.

Covalent organic frameworks (COFs) have recently received increasing interest due to their intriguing properties such as low density, high porosity, good crystallinity and the possibility to introduce a range of organic functional moieties into chemically stable frameworks.^1^–^5^ In terms of photochemistry, this combination of π-conjugated rigid architectures with high porosities and tailorable chemical functionalities in the COF backbones can enable: (a) rapid diffusion of charge carriers in the framework and to the surface, (b) immobilization of organic functionalities to enhance the life-time of excited species by preventing the formation of dimers, and (c) a high interface between the solid COF and reactants, electrolytes, sacrificial donors and co-catalysts. In two-dimensional (2D) COFs, π-functional organic building blocks can be organized in particular stacking patterns, thus that charge carriers can be transported in discrete and periodic columns.^6^ These unidirectional charge carrier pathways can improve the charge carrier mobility and lifetime in 2D COFs.^7^,^8^ Due to these properties COFs have been described as highly promising materials for efficient and recyclable photocatalysis, recently,^9^–^14^ beside other applications, for example for gas adsorption and separation, heterogeneous catalysis, proton conductivity, optoelectronics and energy storage.^15^–^23^

To further improve the catalytic activities, strategies such as introduction of heteroatoms (N and S),^10^ increasing the catalyst wettability,^24^ immobilization of molecular co-catalysts^25^ and improving the conjugation have been endeavored.^26^ It has further been demonstrated that the precise combination of donor–acceptor moieties in organic materials and COFs can yield enhanced performance in solar cells and optoelectronic applications, respectively.^27^–^34^ Herein, two novel COFs are presented, whose structures were designed considering the above-mentioned criteria. These materials have been successfully applied as photoinitiators for efficient and recyclable radical polymerization, paving the way to the development of robust and heterogeneous systems for photochemistry that offers the transfer of radicals induced by visible light.

Considering the beneficial properties of donor–acceptor based materials for charge generation, mobility and stability, we have attempted the synthesis of donor–acceptor COFs as photoinitiators for visible light induced free radical polymerization (Fig. 1).^35^,^36^ TTT–DTDA and TTT–BTDA COFs have been synthesized by the solvothermal reaction of 4,4′,4′′-(1,3,5-triazine-2,4,6-triyl)trianiline (TTT, 106 mg, 0.3 mmol) with thieno[3,2-*b*]thiophene-2,5-dicarbaldehyde (DTDA, 89 mg, 0.45 mmol) or [2,2′-bithiophene]-5,5′-dicarbaldehyde (BTDA, 99 mg, 0.45 mmol), respectively, in presence of 6 M acetic acid (0.5 mL) and mesitylene/dioxane (3 mL) as the solvents [Section S2, ESI†]. In order to synthesize crystalline and porous COFs, a series of experiments was carried out to optimize the best solvent combinations (Tables S1 and S2, ESI†), showing that choosing the right solvent ratio plays a vital role in reaching optimum crystallinity and porosity. The as-synthesized COFs were further characterized by powder X-ray diffraction (PXRD), ^13^C solid-state cross-polarization magic angle spinning nuclear magnetic resonance (CP/MAS-NMR) spectroscopy, N_2_ sorption analyses, scanning electron microscopy (SEM), *in situ* as well as *ex situ* electron paramagnetic resonance (EPR) spectroscopy, and ultraviolet-visible spectroscopy (UV-vis) analyses.

**Fig. 1:**
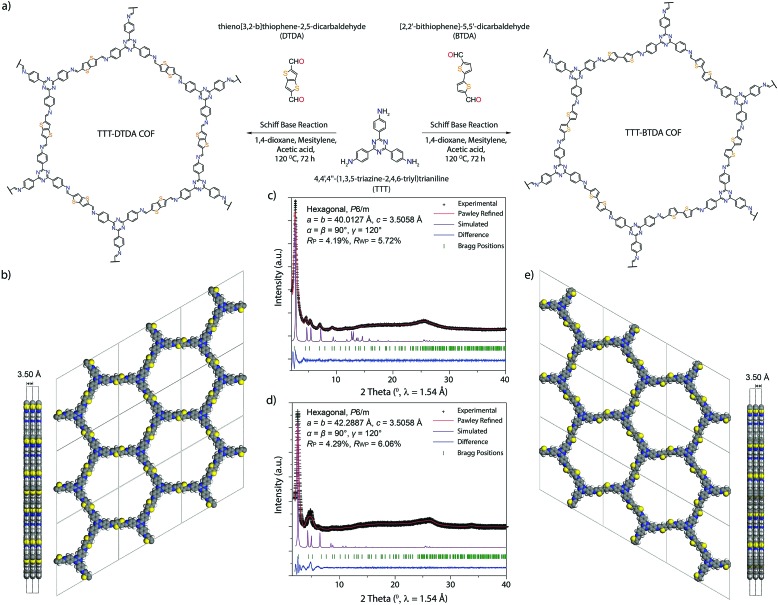
Synthesis and PXRD analyses of TTT–DTDA and TTT–BTDA COFs. (a) Scheme of synthesis of TTT–DTDA and TTT–BTDA following the solvothermal method. (b) Side and top view of the ideal eclipsed (AA) structure of TTT–DTDA. (c and d) Experimental, Pawley-refined and simulated powder X-ray diffraction patterns and a difference plot for TTT–DTDA and TTT–BTDA, respectively. (e) Side and top view of the ideal eclipsed (AA) structure of TTT–BTDA (color: purple, carbon; blue, nitrogen; yellow, sulphur; off-white, hydrogen).

Powder X-ray diffraction (PXRD) analyses with Cu Kα radiation (*λ* = 1.5418 Å) was performed to assess the crystallinity of the as-synthesized COFs. The PXRD patterns for TTT–DTDA and TTT–BTDA are dominated by an intense reflection in the low-angle region, at 2.68 and 2.52 2*θ* degrees for TTT–DTDA and TTT–BTDA, respectively, which can be assigned to the (100) facet of a primitive hexagonal lattice (Fig. 1c and d). Additionally, the presence of further weak reflections and a broad reflection at ∼25 2*θ* degrees for TTT–DTDA and TTT–BTDA COFs that can be assigned to the (001) facet, confirms the formation of two-dimensional (2D) COFs in a crystalline and π–π stacked form. The structural models for TTT–DTDA and TTT–BTDA were constructed by generating the probable 2D hexagonal layers with **hcb** topology with either eclipsed (AA) or staggered (AB) stacking arrangement (Fig. S13, S14 and Section S7, ESI†). After geometrical optimization of the models for TTT–DTDA and TTT–BTDA, the simulated PXRD patterns were calculated and compared with the corresponding experimentally measured patterns. A fully eclipsed AA-type stacking was found to be in agreement with the experimental PXRD patterns (Fig. 1c and d; violet curves). In contrast, AB stacking can be discarded on the account of a significant mismatch in intensities of the PXRD patterns (Fig. S15, ESI†). The final unit cell parameters were determined after Pawley refinement, which led to satisfactorily low residual values and acceptable profile differences (Fig. 1c and d).

The chemical structure of TTT–DTDA and TTT–BTDA was further verified by solid-state NMR spectroscopy. Fig. 2a shows the ^13^C CP/MAS NMR spectrum for TTT–DTDA and TTT–BTDA, where a peak at ∼147 ppm can be assigned to the carbon atoms of the C=N bonds, confirming the condensation reaction of aldehyde (TTT) and primary amines (DTDA or BTDA). The signals at ∼110, 125, 130 and 138 ppm can be ascribed to the carbon atoms of the phenyl groups, while the sharp peak at ∼165 ppm is attributed to the carbon atoms of core triazine ring from TTT linkers in the COFs, validating the presence of intact triazine moieties in the backbones. The formation of frameworks in TTT–DTDA and TTT–BTDA was further corroborated by Fourier transform infrared (FT-IR) spectroscopy. In the FT-IR spectra the presence of a strong vibration at 1582 cm^–1^ can be assigned to the stretching mode of the –C=N bonds (Fig. S4, ESI†), indicating the formation of imine bonds in COFs. Meanwhile, the aldehyde (∼1645 cm^–1^) and amino (∼3450 cm^–1^) stretching bands of respective starting materials completely disappeared after COF formation (Fig. S4, ESI†), which offers further evidence for the formation of imine bonds *via* the condensation of aldehyde and primary amines.

**Fig. 2:**
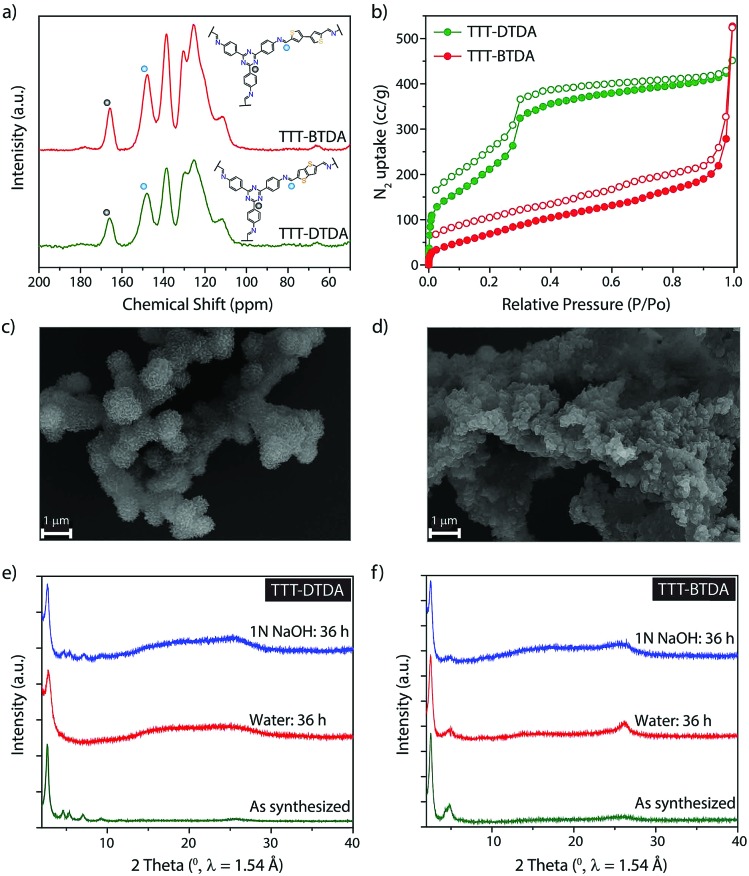
Characterization and chemical stability of COFs. (a) ^13^C CP-MAS solid-state NMR spectroscopy analyses of TTT–DTDA and TTT–BTDA. Inset image shows the structures of these COFs. (b) N_2_ adsorption–desorption isotherms for TTT–DTDA and TTT–BTDA. (c) SEM image of TTT–DTDA and (d) TTT–BTDA. (e and f) PXRD patterns of TTT–DTDA and TTT–BTDA after 36 h treatment in water and 1 N NaOH compared with as-synthesized samples.

The permanent porosity of TTT–DTDA and TTT–BTDA was evaluated by N_2_ adsorption–desorption isotherm measured at 77 K (Fig. 2b). The calculated Brunauer–Emmett–Teller (BET) and Langmuir surface area values for TTT–DTDA were 1012 and 2195 m^2^ g^–1^, respectively, whereas TTT–BTDA showed lower BET (302 m^2^ g^–1^) and Langmuir (440 m^2^ g^–1^) surface areas. In case of TTT–BTDA, most probably, due to the partial offset stacking of adjacent layers seen in the less defined PXRD pattern in comparison to TTT–DTDA, just moderate surface area values were observed (Fig. S3, ESI†).^37^–^39^ Scanning electron microscopy (SEM) analyses indicate that TTT–DTDA and TTT–BTDA crystallize in spherical and flower-like crystallites, respectively (Fig. 2c, d and S5, ESI†). The thermal stability of as-synthesized TTT–DTDA and TTT–BTDA was evaluated by thermogravimetric analyses, showing no weight loss under nitrogen atmosphere up to 450 °C (Fig. S5, ESI†). Further, to analyze the chemical stabilities of COFs, 50 mg of TTT–DTDA and TTT–BTDA were submerged in water and 1 N NaOH. The samples were kept immersed in the respective liquids for 36 h and after washing with water, their structure was analyzed by PXRD. Both COFs retained their crystalline structure in aqueous and alkaline media (Fig. 2e and f). Besides, the sample could be completely recovered in quantitative yield after the chemical treatment, thus showing no indication of decomposition, even though some loss in surface area was observed (Fig. S12, ESI†). Obviously, the stability of the COFs in water and alkaline media is an important prerequisite for their application in visible light induced radical polymerization that generally takes place in presence of tertiary amines as initiators.^35^,^36^,^40^,^41^

Photopolymerization, a process in which visible or ultraviolet light initiates and propagates a polymerization reaction to form linear or cross-linked polymer structures, is an important process in polymer chemistry due to its wide range of economic and ecological applications.^42^,^43^ This process has been widely used for coatings, adhesives, microelectronics, optical waveguides, *etc.*^43^,^44^ In order to meet the industrial requirements of products obtained from the photopolymerization process, a variety of photoinitiators have been established and applied successfully.^45^,^46^ However, most of these low molecular weight photoinitiators suffer from severe drawbacks such as migration from cured films, high volatility and strong odor. To address these concerns and achieve a higher durability of the catalysts, heterogeneous photoinitiators like polymeric carbon nitride,^35^ porous polymers^36^ metal organic frameworks (MOFs),^47^ onium salts,^48^ cross-linked polymers,^49^,^50^ *etc.* have been exploited. Although these materials show promising results regarding recyclability, lower activities of these heterogeneous visible-light photoinitiators have been observed in comparison to their molecular counterparts, probably due to their low surface areas and/or less accessible reactive sites. Considering the significant porosity and high surface areas, and the presence of donor–acceptors moieties in the frameworks allowing facile migration of photogenerated charge carriers, the performance of TTT–DTDA and TTT–BTDA have been investigated for visible-light induced, heterogeneous free radical polymerization.

EPR spectra of both COFs have been recorded in the dark and under photocatalytic reaction conditions to visualize charge separation and transfer. Both COFs show a narrow isotropic singlet with Lorentzian line shape at *g* = 2.005. This signal increased when TTT–DTDA and TTT–BTDA samples were irradiated with light, since more electrons are excited from the valence to the conduction band, indicating electron–hole pair generation in the COF semiconductor (Fig. 3a and b, green spectra).^51^–^53^ After switching off the light, the EPR signal intensity decreased again, due to recombination of the CB-e^–^ with holes (Fig. 3a and b, red spectra).^54^ The drop of the EPR signal is more pronounced for the less active sample TTT–BTDA, suggesting that undesired recombination of CB-e^–^ with holes is faster than transfer of the latter to potential reactants.

**Fig. 3:**
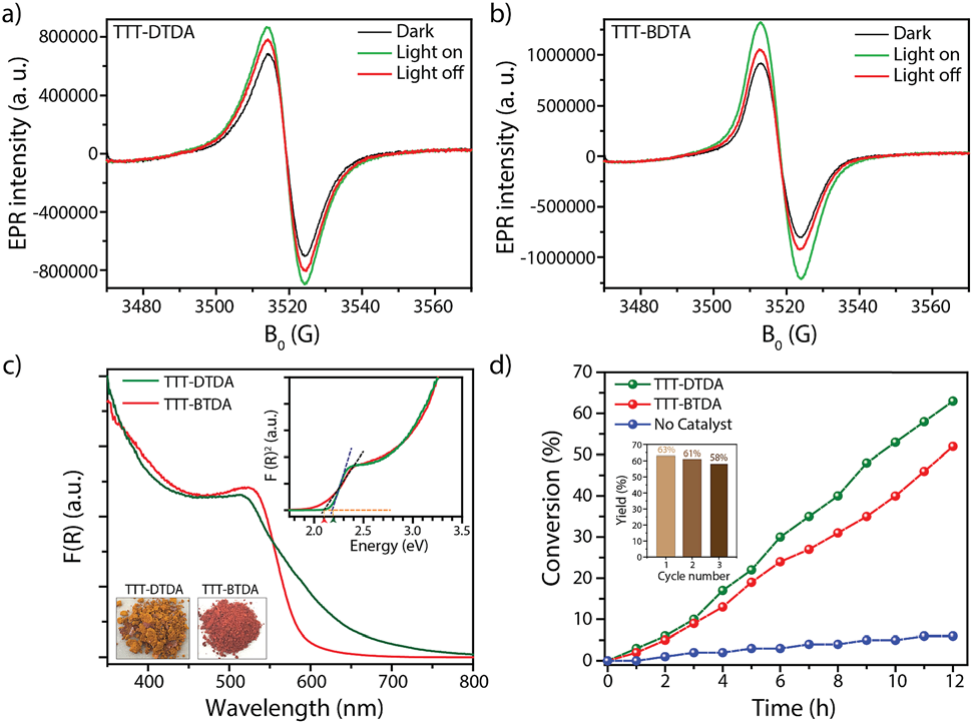
(a and b) EPR CB e^–^ signals of TTT–DTDA and TTT–BTDA under dark condition (black), during visible light irradiation (green) and after switching off the light (red). (c) UV-vis diffuse reflectance spectra of TTT–DTDA and TTT–BTDA. The inset shows Tauc plots and optical images of the COF powders. (d) Photo-polymerization of MMA using TTT–DTDA and TTT–BTDA, in presence of triethylamine at room temperature (argon atmosphere). Inset image shows the recyclability of TTT–DTDA for radical polymerization for three cycles.

During irradiation with UV-vis light, a radical signal developed in the *operando* EPR spectra (exemplarily shown for TTT–DTDA in Fig. 4a, for comparison of both samples see Fig. S8, ESI†) and polymer formation was observed with time inside the EPR flat cell for both catalysts (Fig. S9, ESI†). This signal can be assigned to the propagating radical –C_β_H_2_–˙C_α_(CH_3_)–COOCH_3_. The signal recorded after 160 min reaction time (Fig. 4a) could be satisfactorily reproduced by spectra simulation using *g* = 2.0048 and superhyperfine structure (shfs) constants for the coupling of the electron at C_α_ with the three CH_3_ protons and the two protons at C_β_, *A*_CH_3__ = 22.2 G, *A*_H_β1__ = 14.7 G, *A*_H_β2__ = 7.9 (Fig. S9a, ESI†), which are in good agreement with literature data.^55^,^56^ This species might be produced upon reaction of MMA with initiating radicals formed by transfer of holes from COF to TEA (Fig. 4b). Notably, such TEA-derived radicals have not been seen in the operando EPR spectra, probably due to fast reaction with MMA and/or too low concentration.^53^ Diffuse reflectance UV-vis spectra of TTT-DTDA and TTT-BTDA exhibit a broad and strong absorbance peak in the visible region and an absorbance edge around 527 and 515 nm, respectively (Fig. 3c). As compared with TTT-BTDA, the absorbance tail for TTT-DTDA is extended beyond 750 nm, yielding enhanced light absorption. The calculated optical band gaps from Tauc plots are 2.10 and 2.19 eV, respectively, for TTT-BTDA and TTT-DTDA (Fig. 3c, inset).

**Fig. 4:**
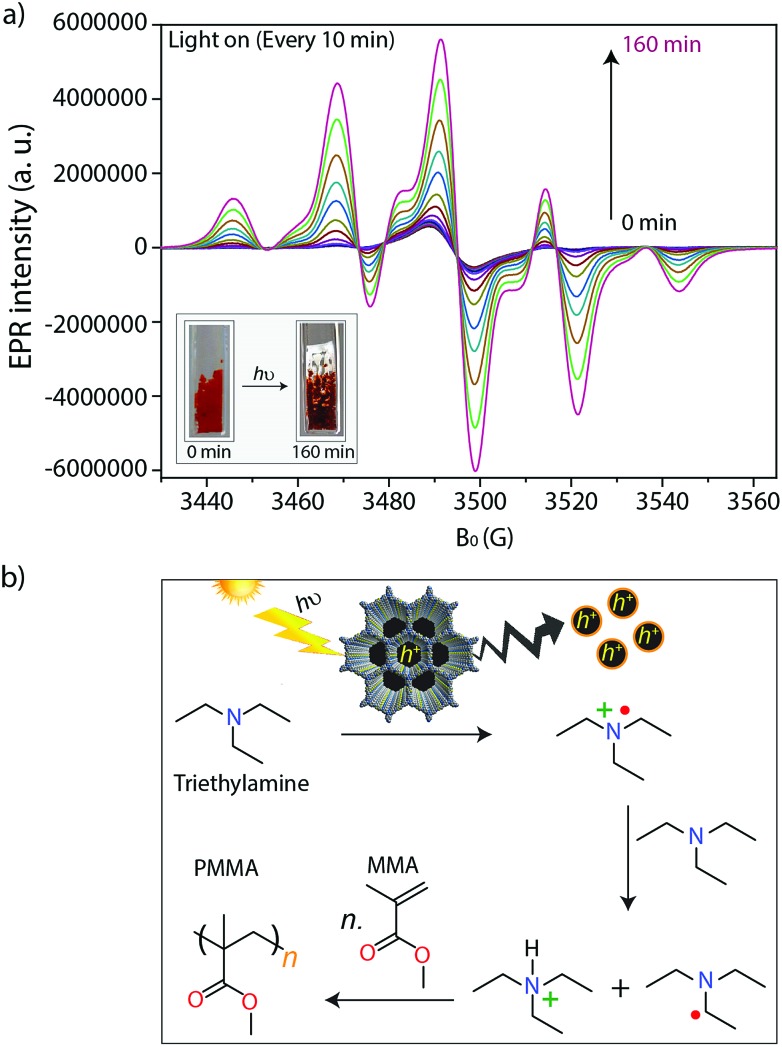
(a) *In situ* EPR spectra measured every 10 minutes during light irradiation. Reaction conditions: 5 mg TTT–DTDA (COFs), 0.25 mL methyl methacrylate (MMA), 12.5 μL triethylamine (TEA) and UV-vis light irradiation using a 300 W Xe lamp with an output power 1.5 W. The inset image shows the light induced conversion of MMA to PMMA in the flat cell. (b) Proposed mechanism for free radical polymerization of MMA to PMMA using porous COFs, in presence of triethylamine as a co-initiator.

Considering the light absorption and charge separation under visible light irradiation, visible light induced radical polymerization experiments using the as-synthesized COFs were attempted, using a water-cooled glass reactor equipped with stirring plate and external light source (*λ* > 420 nm) (Fig. S1, ESI†). A mixture of methyl methacrylate (MMA; 1 mL, 9.39 mmol) and triethylamine (TEA; 35 mg, 0.35 mmol) was irradiated with light in presence of the respective COFs (20 mg). TEA acts as co-initiators for radical polymerization reaction (Section S2 and Table S3, ESI†). During the whole process, the temperature was maintained at 25 °C and the reaction was performed under argon atmosphere. After performing the reaction for a given time, the reaction mixture was dissolved in THF, the COFs were separated by filtration and the remaining solutes were re-precipitated in methanol to obtain poly-methyl methacrylate (PMMA). The polymeric products were dried at room temperature. In order to analyze the performance of TTT–DTDA and TTT–BTDA COFs for radical polymerization, the MMA conversion *vs.* time was plotted (Fig. 3d). TTT–DTDA showed the highest conversion up to 63% after 12 h of reaction time, whereas TTT–BTDA showed a lower activity to yield ∼54% conversion. When the same reaction was carried out without COF as catalyst, just negligible conversions were observed (Fig. 3b). Notably, the COFs showed a better performance in radical polymerization of MMA to PMMA with higher molecular weight (Table 1), compared with carbon nitride,^35^ porous polymers^36^ MOFs,^47^ cross-linked polymers,^49^,^50^ *etc.*,^43^ probably due to the combination of their high surface area and π-conjugated, donor–acceptor type backbones (Table S6, ESI†).

**Table 1:** Bulk polymerization of methyl methacrylate initiated using visible light in presence of TTT–DTDA and TTT–BTDA COFs and triethylamine as co-initiators, at room temperature^*a*^

COF	Conversion (%)	Molecular wt (*M*_n_) (g mol^–1^)	Dispersity^*b*^ *M*_w_/*M*_n_
TTT–DTDA	63%	2.3950 × 10^5^	2.2161
TTT–BTDA	54%	2.4292 × 10^5^	2.9246
No catalyst^*c*^	5%	0.9437 × 10^2^	1.1328

^*a*^ *λ* = 420–700 nm, time = 12 h, [MMA] = 9.39 mmol, [TEA] = 0.35 mmol, 20 mg of as-synthesized COF initiator.

^*b*^Number-average molecular weight (*M*_n_) and molecular weight distribution (*M*_w_/*M*_n_) determined by GPC.

^*c*^Without any addition of COF initiator.

Most importantly, due to the heterogeneous nature, the COF-based photoinitiators maintain its photoinitiation activity, thus can be easily separated and used for further polymerizations with nearly maintained activities (Fig. 3d, inset). After the photopolymerization experiments, TTT–DTDA and TTT–BTDA samples were recovered, thoroughly washed, and then again characterized by ^13^C CP-MAS solid-state NMR spectroscopy analyses, showing the preservation of the COF structure (Fig. S10, ESI†). The PXRD analyses of the recovered COFs showed that the materials maintained their crystallinity, confirming the robustness of COF-based photoinitiators for radical polymerization (Fig. S11, ESI†). In the control experiments, the reaction without initiator (COF) and co-initiators (TEA) yielded no or just a negligible amount of product, suggesting the necessity of both COF and TEA for radical polymerization (Table S3, ESI†). In addition, no polymer was obtained without light irradiation or in the presence of oxygen (O_2_) suggesting a light-driven process as well as involvement of a radical polymerization mechanism (Table S3, ESI†).^42^ In additional control experiments, the radical polymerization was carried out using an amorphous TTT–DTDA polymer, a simple mixture of the monomers, and another COF with comparable structure but without the strong electron donating thiophenes in the backbone (IISERP-COF4),^57^ which all yielded lower conversions of 42%, 37% and 7%, respectively (Fig. S16 and Table S3, ESI†). These results prove the importance of the open, crystalline, donor–acceptor type structure of the COF for enhancing its photoinitiating activity for free radical polymerization.

After the absorption of light in the visible region by the COFs, the photochemically formed holes can oxidize triethylamine to the corresponding radical cations, which abstract hydrogens from another amine molecule leading to the formation of amine radicals, which subsequently are starting the radical polymerization reaction with MMA (Fig. 4b).^35^,^36^,^47^,^58^ The formation of radicals and their relation to catalytic activity have been confirmed by *in situ* EPR analysis (Fig. 4a and S7, ESI†). In these experiments, we have observed that undesired electron–hole pair recombination is slower for catalyst TTT–DTDA, while the radical formation is most pronounced for this catalyst. Both aspects might be responsible for the higher catalytic activity of this COF in comparison to TTT–BTDA.

## Conclusions

In summary, we have synthesized two donor–acceptor COFs using electron deficient triazine building block and electron rich thiophene-based linkers. The crystalline and highly porous COFs absorb light in the visible region. The as-synthesized COFs, having promising photochemical properties, have been utilized to initiate the free radical polymerization of methyl methacrylate (MMA) to poly-methyl methacrylate (PMMA), where COFs play a key role in the generation of holes that induce hydrogen abstraction from the amine co-initiator. The facile separation of these COF-photoinitiators from the MMA/PMMA mixture and their recyclability for multiple radical polymerization cycles together with their capability to initiate radical reactions under visible-light, make these materials very promising photoinitiators for a range of photochemical reactions.

## Conflicts of interest

There are no conflicts to declare.

## Supplementary Material

Supplementary information
