# Supplementary material for: Donor–acceptor covalent organic frameworks for visible light induced free radical polymerization
Source: Chem Sci. 2019 Aug 5;10(36):8316–22. doi: 10.1039/c9sc02601k (PMC6855310; doi:10.1039/c9sc02601k)
Supplement: Supplementary file 1 [file SC-010-C9SC02601K-s001.pdf]

Electronic Supplementary Material (ESI) for Chemical Science.
This journal is © The Royal Society of Chemistry 2019

Supporting Electronic Supplementary Material (ESI) for
Chemical Science

This journal is © The Royal Society of Chemistry 2019

Donor-acceptor Covalent Organic Frameworks for visible light induced free radical polymerization

Pradip Pachfule,*[a] Amitava Acharjya,[a] Jérôme Roeser,[a] Ramesh P. Sivasankaran,[b] Mengyang Ye,[a] Angelika Brückner,[b] Johannes Schmidt[a] and Arne Thomas*[a]

[a] *Department of Chemistry/Functional Materials, Technische Universität Berlin, Hardenbergstraße 40, 10623 Berlin, Germany.*

E-mail: *pradip.pachfule@campus.tu-berlin.de; arne.thomas@tu-berlin.de*

[b] *Leibniz Institute for Catalysis, University of Rostock, Albert-Einstein-Str. 29a, 18059 Rostock, Germany.*

Abstract: Covalent organic frameworks (COFs) are promising materials for applications in photocatalysis, due to their conjugated, porous and chemically stable architectures. Alternating electron Donor-Acceptor-type structures are known to enhance charge carrier transport mobility and stability in polymers and are therefore also interesting building units for COFs used as photocatalysts but also as photoinitiator. In this work, two donor-acceptor COFs using electron deficient *4,4',4''*-(1,3,5-triazine-2,4,6-triyl)trianiline and electron rich thiophene-based thieno[*3,2-b*]thiophene-2,5-dicarbaldehyde or [2,2'-bithiophene]-5,5'-dicarbaldehyde linkers are presented. The resulting crystalline and porous COFs have been applied as photoinitiator for visible light induced free radical polymerization of methyl methacrylate (MMA) to poly-methyl methacrylate (PMMA). These results pave the way to the development of robust and heterogeneous systems for photochemistry that offers the transfer of radicals induced by visible light.

DOI: 10.1039/x0xx00000x

Table of Contents

Section S1. Materials and methods....3

Section S2. Synthesis of COFs and details about the radical polymerization reaction....6

Section S3. Pore size distribution for COFs....9

Section S4. Fourier transform infrared and scanning electron microscopy analyses....10

Section S5. Thermogravimetric analyses (TGA) and EPR spectroscopy analyses for COFs....12

Section S6. Structure modeling and atomic coordinates of COFs. 13

Section S7. COF stability after radical polymerization of MMA to PMMA.. 15

Section S8. COF stability after radical polymerization of MMA to PMMA.. 22

Section S9. References....24

Author Contributions....24

Section S1. Materials and methods:

General

All inert reactions and manipulations were carried out in an argon atmosphere using standard Schlenk techniques or in an MBRAUN (MB-120 BG) inert atmosphere glove box containing an atmosphere of argon. Unless otherwise specified, reagents were used as received without further purification.

Materials

Anhydrous 1,4-dioxane (99.9%), mesitylene (99 %) and triethylamine (≥99.5%) were purchased from Sigma-Aldrich. Methacrylic acid methyl ester (MMA) (>99 %), methanol (99 %), tetrahydrofuran (99 %), acetone (>99 %) and acetic acid (98 %) were purchased from Carl Roth. 2,2'-Bithiophene-5,5'-dicarboxaldehyde (98 %) and Thieno[3,2-b]thiophene-2,5-dicarboxaldehyde (>93 %) were purchased from TCI Chemicals. 4,4',4''-(1,3,5-Triazine-2,4,6-triyl)trianiline (>98 %) was purchased from Ark Pharm.

NMR measurements

Liquid-state ^{1}H NMR and ^{13}C NMR were recorded on a Bruker Avance II 200 and Bruker Avance 400 MHz spectrometer in the given solvent. $^{13}C\{^{1}H\}$ cross polarized magic angle spinning (CP MAS) NMR measurements were carried out using a Bruker range Avance 400 MHz Solid State spectrometer operating at 100.6 MHz for ^{13}C and a Bruker 4 mm double resonance probe-head operating at a spinning rate of 10 kHz.

Physisorption measurements

Nitrogen (N_2) sorption analyses were conducted at 77 K using an Autosorb-iQ-MP from Quantachrome. The pore size distributions were calculated from the adsorption isotherms by Quenched Solid State Functional Theory (QSDFT) using N_2 sorption data collected at 77 K. We used the carbon slit/cylindrical/spherical pore model for analyzing the distribution. Before analysis, samples were degassed at 150 °C for 24 h. BET and Langmuir surface areas were determined over a P/P_0 range of 0.1-0.25.

Thermogravimetric analysis

TGA measurements were carried out under nitrogen atmosphere on a Mettler Toledo TGA 1 Stare thermal instrument with a heating rate of 10 K min^{-1}.

X-ray photoelectron spectroscopy

X-Ray photoelectron spectra were measured on a K-Alpha™ + X-ray Photoelectron Spectrometer System (Thermo Scientific) with Hemispheric 180° dual-focus analyzer with 128-channel detector. Micro focused Al-K*α* radiation was used as X-ray monochromator.

Other Characterizations:

Powder X-ray diffraction (PXRD) data was collected on a Bruker D8 Advance diffractometer in reflection geometry operating with a Cu K*α* anode (λ = 1.54178 Å) operating at 40 kV and 40 mA. Samples were ground and mounted as loose powders onto a Si sample holder. PXRD patterns were collected from 2 to 60 *2θ* degrees with a step size of 0.02 degrees and an exposure time of 2 seconds per step. The Fourier transform infrared spectroscopy (FT-IR) analyses of the samples were carried on Varian 640IR spectrometer equipped with an ATR cell. Solid-state diffuse reflectance Ultraviolet–visible spectroscopy (UV-*vis*) spectra of the as synthesized COF powders have been collected on Varian Cary 300 UV-*Vis* Spectrophotometer. The scanning electron microscope (SEM) analyses of COF samples were performed on S-2700 scanning electron microscope (Hitachi, Tokyo, Japan).

Electron paramagnetic resonance (EPR) spectroscopy studies:

EPR measurements in X-band (microwave frequency ≈ 9.8 GHz) were performed at 300 K by a Bruker EMX CW-micro spectrometer equipped with an ER 4119HS-WI high-sensitivity optical resonator with a grid in the front side. The samples were illuminated by a 300 W Xe lamp with 420 nm cut-off filter (LOT Oriel). All the samples were measured under the same conditions (microwave power: 6.99 mW, receiver gain: 1×10^4, modulation frequency: 100 kHz, modulation amplitude: 3 G, Sweep time: 122.8 s). g values have been calculated from the resonance field B_0 and the resonance frequency v using the resonance condition $h\nu = g\beta B_0$. The calibration of the g values was performed using DPPH (2,2-diphenyl-1- picrylhydrazyl) (g = 2.0036 ± 0.00004). For operando measurements under photocatalytic reaction conditions, a special quartz flat cell was used. Spectra simulation was performed with the program Simphonia (Bruker).

Radical polymerization of methyl methacrylate (MMA) to poly-methyl methacrylate (PMMA)*[1,2]*:

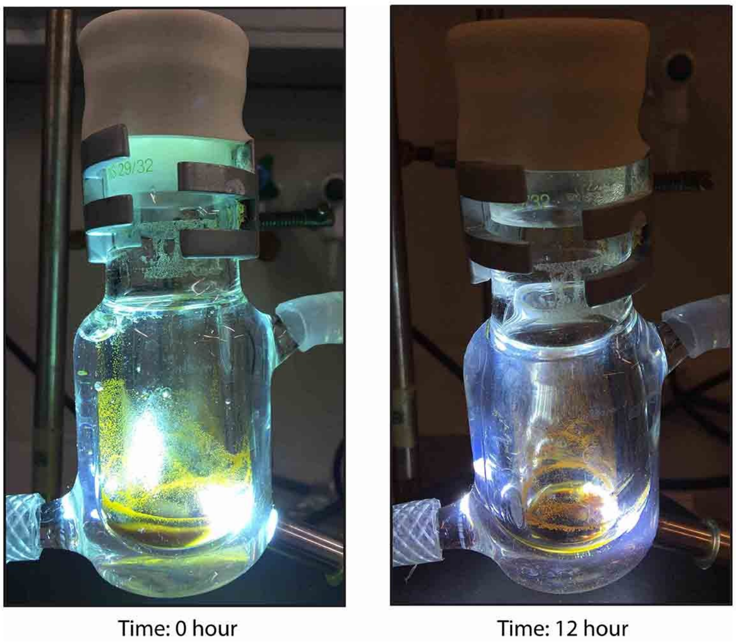

Figure S1. *The temperature-controlled photo-reactor used for radical polymerization of MMA to PMMA. The left image shows the reaction mixture at the start of photocatalysis, whereas image at right shows the almost solidified mixture after 12 hours.*

The radical polymerization reaction of MMA to PMMA, initiated using COFs as initiator and triethylamine (TEA) as co-initiator, was performed using glass reactor as shown in Figure S1. The addition of MMA, TEA and COF initiator into glass reactor has been done in the glove box, operating under Argon atmosphere. The temperature of reaction mixture was maintained at 25 °C using external water circulator, whereas the mixture was stirred using an external magnetic stirrer. The Lumatec Superlite 400 (500 W mercury lamp) equipped with various filters and variable light intensity has been used as light source for performing the radical polymerization reaction. The wavelength (λ) of 400-700 nm was used for reaction, with 75% light intensity.

Section S2. Synthesis of COFs and details about the radical polymerization reaction:

Synthesis of TTT-DTDA and TTT-BTDA COFs:

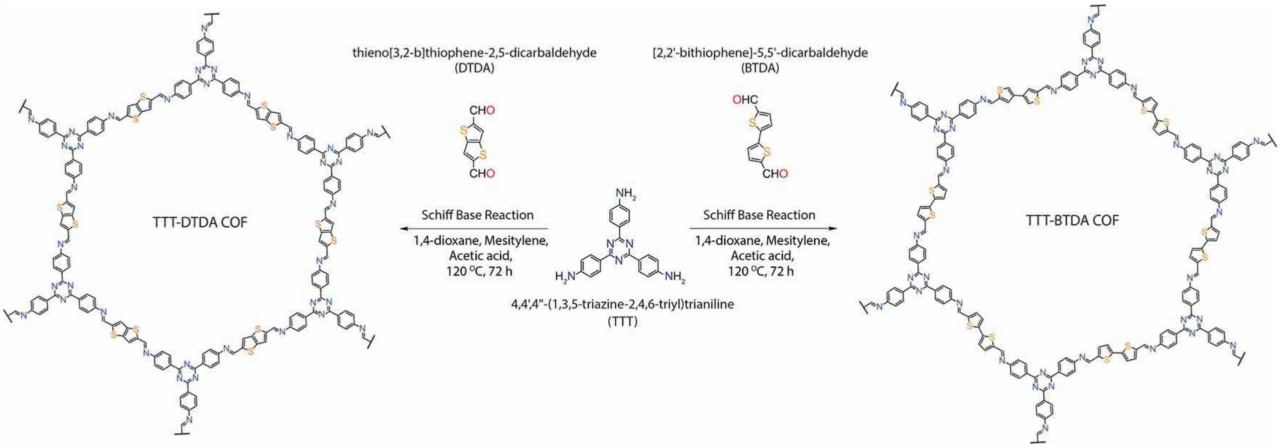

Figure S2. *Synthesis of TTT-DTDA and TTT-BTDA COF using solvothermal method. The reaction of 4,4',4''-(1,3,5-triazine-2,4,6-triyl)trianiline and thiophene-based thieno[3,2-b]thiophene-2,5-dicarbaldehyde or [2,2'-bithiophene]-5,5'-dicarbaldehyde linkers yielded TTT-DTDA and TTT-BTDA COFs, respectively, in presence of acetic acid as catalyst.*

Synthesis of TTT-DTDA COF:

A pyrex tube (o.d. × i.d. = 18 × 14 mm^2 and length 10 cm) was charged with 4,4',4''-(1,3,5-triazine-2,4,6-triyl)trianiline (TTT, 106 mg, 0.3 mmol), thieno[3,2-*b*]thiophene-2,5-dicarbaldehyde (DTDA, 89 mg, 0.45 mmol), 1.5 mL of mesitylene, 1.5 mL of dioxane and 0.5 mL of 6 M aqueous acetic acid. The whole content was sonicated for 10 minutes in order to get a homogenous dispersion. The tube was then flash frozen at 77 K (liquid N_2 bath) and degassed by three freeze-pump-thaw cycles. The tube was sealed off under vacuum and then heated at 120 °C for 3 days. A brown colored precipitate was collected by centrifugation, washed with anhydrous methanol and finally with anhydrous acetone. The powder collected was then purified by soxhlet treatment with methanol (12 h) at 90 °C, and then dried at 100 °C for 2 hours to yield a brown colored pure TTT-DTDA powder.

Isolated yield = ~80% (155 mg).

Synthesis of TTT-BTDA COF:

A pyrex tube (o.d. × i.d. = 18 × 14 mm^2 and length 10 cm) was charged with 4,4',4''-(1,3,5-triazine-2,4,6-triyl)trianiline (TTT, 106 mg, 0.3 mmol), [2,2'-bithiophene]-5,5'-dicarbaldehyde (BTDA, 99 mg, 0.45 mmol), 1.0 mL of mesitylene, 2.0 mL of dioxane and 0.5 mL of 6 M aqueous acetic acid. The whole content was sonicated for 10-12 minutes in order to get a homogenous dispersion. The tube was then flash frozen at 77 K (liquid N_2 bath) and degassed by three freeze-pump-thaw cycles. The tube was sealed off under vacuum and then heated at 120 °C for 3 days. A red colored precipitate was collected by centrifugation, washed with anhydrous methanol and finally with anhydrous acetone. The powder collected was then purified by soxhlet treatment with THF (12 h) at 90 °C, and then dried at 100 °C for 2 hours to give a dark-red colored pure TTT-BTDA powder.

Isolated yield = ~55% (112 mg).

Synthetic Conditions for COF Synthesis:

Table S1. *Optimization of the reaction conditions for TTT-DTDA COF:*

TTT-DTDA COF:

TTT = 106 mg (0.3 mmol), DTDA = 89 mg (0.45 mmol), Acetic acid = 0.5 ml (6 M).

Serial No.	Solvent combination	Product observed? Yes/No	Crystallinity
1	Methanol (1.5 mL), Mesitylene (1.5 mL)	Yes	No crystallinity
2	Methanol (2.0 mL), Mesitylene (1.0 mL)	Yes	No crystallinity
3	1,4-dioxane (1.0 mL), Mesitylene (2.0 mL)	Yes	No crystallinity
4	1,4-dioxane (1.5 mL), Mesitylene (1.0 mL)	Yes	Low crystallinity
5	1,4-dioxane (1.5 mL), Mesitylene (1.5 mL)	Yes	Moderate crystallinity
6	1,4-dioxane (2.0 mL), Mesitylene (1.0 mL)	Yes	High crystallinity

Table S2. *Optimization of the reaction conditions for TTT-BTDA COF:*

TTT-BTDA COF:

TTT = 106 mg (0.3 mmol), BTDA = 99 mg (0.45 mmol), Acetic acid = 0.5 ml (6 M).

Serial No.	Solvent combination	Product observed? Yes/No	Crystallinity
1	Methanol (1.5 mL), Mesitylene (1.5 mL)	Yes	No crystallinity
2	Methanol (2.0 mL), Mesitylene (1.0 mL)	Yes	No crystallinity
3	1,2-Dichlorobenzene (1.5 mL), Mesitylene (1.5 mL)	Yes	No crystallinity
4	1,2-Dichlorobenzene (1.5 mL), *N*,*N*-Dimethylformamide (1.5 mL)	Yes	No crystallinity
5	1,4-dioxane (1.0 mL), Mesitylene (1.5 mL)	Yes	Moderate crystallinity
6	1,4-dioxane (1.5 mL), Mesitylene (1.5 mL)	Yes	High crystallinity

Radical Polymerization Reaction:

A glass reactor, jacketed with external water cooling channel, was charged with 1.0 ml MMA (9.39 mmol), 35 mg of TEA (0.35 mmol) and 20 mg of as-synthesized COF initiator, under argon atmosphere. The magnetic needle was added into the reaction mixture and the reactor was closed with rubber septum for isolation from air. Afterwards, the content was irradiated using a 500 W mercury lamp equipped with a cutoff filter (400-700 nm), 75% intensity and a water cooling system for 12h (Figure S1). At the end of irradiation, the bulk solid was dissolved in THF (10-15 mL), filtered and precipitated in methanol (250 mL). The PMMA polymers were collected by filtration and then dried in air for 24 h. The recovered COF initiator was washed multiple times with THF and then subjected to the soxhlet treatment for 2h to get rid of the PMMA polymer trapped in COF pores.

The similar protocol was followed for using the using the TTT-DTDA and TTT-BTDA initiators. For the reaction without initiator, except COF, all other parameters were kept constant for polymerization reaction. The optimization of conditions for polymerization reaction:

Table S3. *The optimization of reaction conditions for radical polymerization of MMA to PMMA:*

Sr. No.	MMA	Co-initiator	Initiator	Time	Light	Yield
1	1 ml	TEA: 35 mg	20 mg TTT-DTDA	12 h	λ = 420-700 nm	63%
2	1 ml	TEA: 35 mg	20 mg TTT-BTDA	12 h	λ = 420-700 nm	54%
3	1 ml	TEA: 20 mg	20 mg TTT-DTDA	12 h	λ = 420-700 nm	40%
4	1 ml	TEA: 0 mg	20 mg TTT-DTDA	12 h	λ = 420-700 nm	3%
5	1 ml	TEA: 35 mg	10 mg TTT-DTDA	12 h	λ = 420-700 nm	24%
6	1 ml	TEA: 35 mg	No initiator	12 h	λ = 420-700 nm	5%
7[a]	1 ml	TEA: 35 mg	20 mg TTT-DTDA	12 h	No light	0%
8[b]	1 ml	TEA: 35 mg	20 mg TTT-DTDA	12 h	λ = 420-700 nm	2%
9	1 ml	TEOA: 35 mg	20 mg TTT-DTDA	6 h	λ = 420-700 nm	48%
10[c]	1 ml	TEA: 35 mg	20 mg TTT-DTDA	12 h	λ = 420-700 nm	45%
11[d]	1 ml	TEA: 35 mg	20 mg TTT-DTDA Polymer	12 h	λ = 420-700 nm	42%
12	1 ml	TEA: 35 mg	Mixture of 9.9 mg TTT and 11.1 mg DTDA linkers	12 h	λ = 420-700 nm	35%
13[e]	1 ml	TEA: 35 mg	20 mg IISERP-COF4	12 h	λ = 420-700 nm	7%

[a] *Under dark conditions.*

[b] *No argon atmosphere (open flask reaction in atmospheric conditions, in presence of oxygen).*

[c] *Temperature = 0 °C (273 K).*

[d] *Reaction using the TTT-DTDA Polymer prepared following the Schiff-base reaction, using atmospheric conditions.*

[e] *Using the non-donor acceptor (non-DA) type COF IISERP-COF4 synthesized using reported procedure.*[3]

Acronyms: *MMA = methyl methacrylate; TEA = Triethylamine; TEOA = Triethanolamine*

Section S3. Pore size distribution for COFs

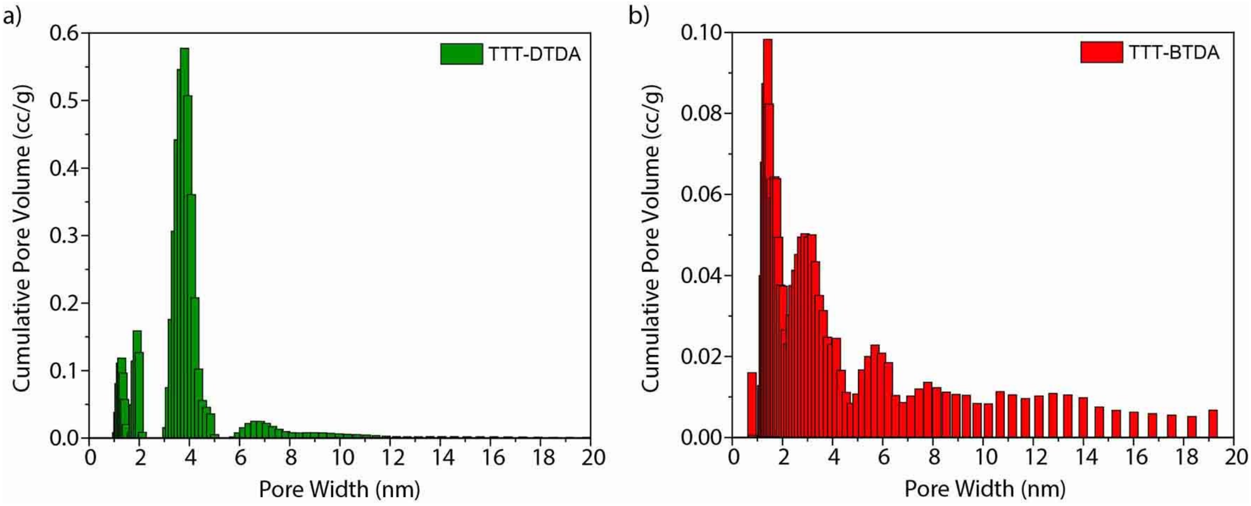

Figure S3. *Pore size distribution for TTT-DTDA and TTT-BTDA COFs, showing the pore distribution very close to the ideal pore size calculated from simulated structures.*

TTT-DTDA COF:

Method = Quenched Solid State Functional Theory (QSDFT)

Model = N_2 at 77 K on carbon slit/cylindrical/spherical pore (Fitting error = 2.5%)

Total Pore Volume: 0.6986 cc/g (at P/P_o = 0.99454)

TTT-BTDA COF:

Method = Quenched Solid State Functional Theory (QSDFT)

Model = N_2 at 77 K on carbon slit/cylindrical/spherical pore (Fitting error = 1.5 %)

Total Pore Volume: 0.8151 cc/g (at P/P_o = 0.99494)

Section S4. Fourier transform infrared (FT-IR) and scanning electron microscopy (SEM) analyses:

FT-IR analyses for COFs:

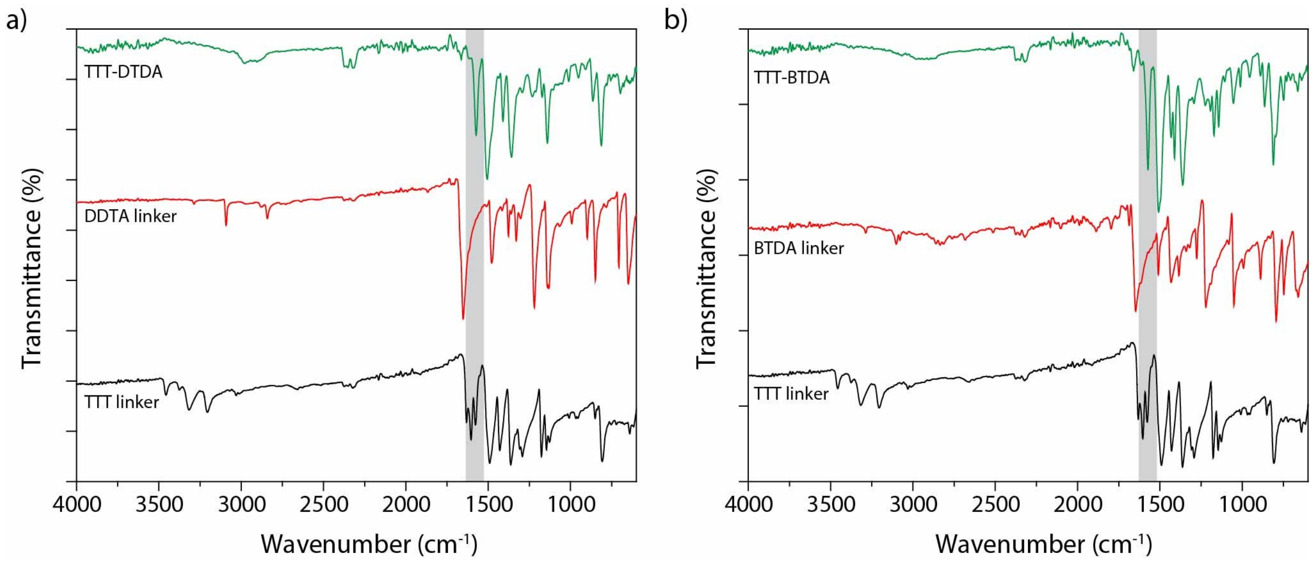

Figure S4. *Fourier transform infrared (FT-IR) spectrum for TTT-DTDA and TTT-BTDA COFs showing a presence of strong vibration at ~1582 cm^{-1} corresponding to the stretching mode of the -C═N bonds indicating the formation of imine bonds in COFs.*[4]

SEM analyses for COFs:

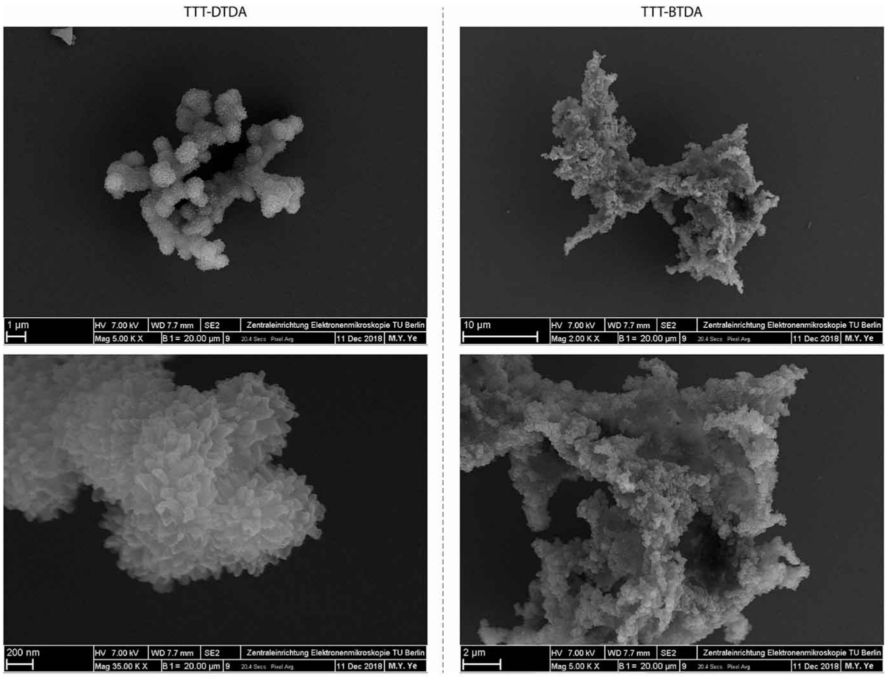

Figure S5. *Scanning electron microscopy (SEM) analyses for TTT-DTDA and TTT-BTDA COFs.*

Section S5. Thermogravimetric analyses (TGA) and Electron paramagnetic resonance (EPR) spectroscopy analyses for COFs:

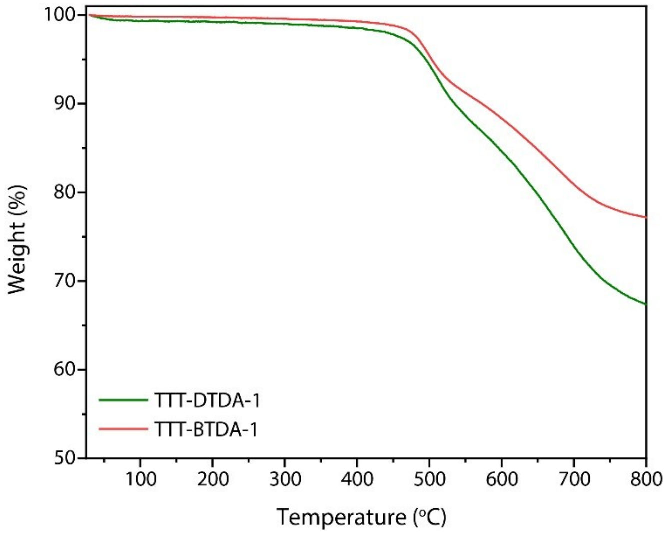

Figure S6. *Thermogravimetric analyses (TGA) for TTT-DTDA and TTT-BTDA COFs, showing the thermal stability of COFs beyond 450 °C, under nitrogen atmosphere.*

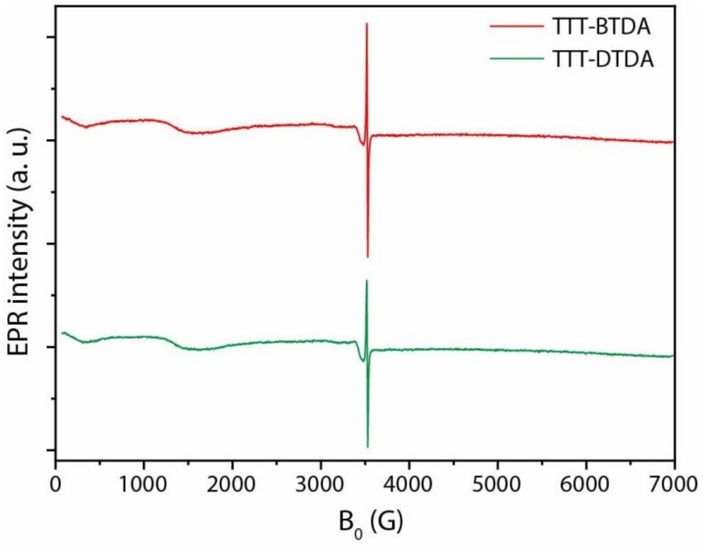

Figure S7. *EPR measurements for TTT-DTDA and TTT-BTDA COFs collected at 300 K, illuminating the samples by a 300 W Xe lamp with 420 nm cut-off filter.*

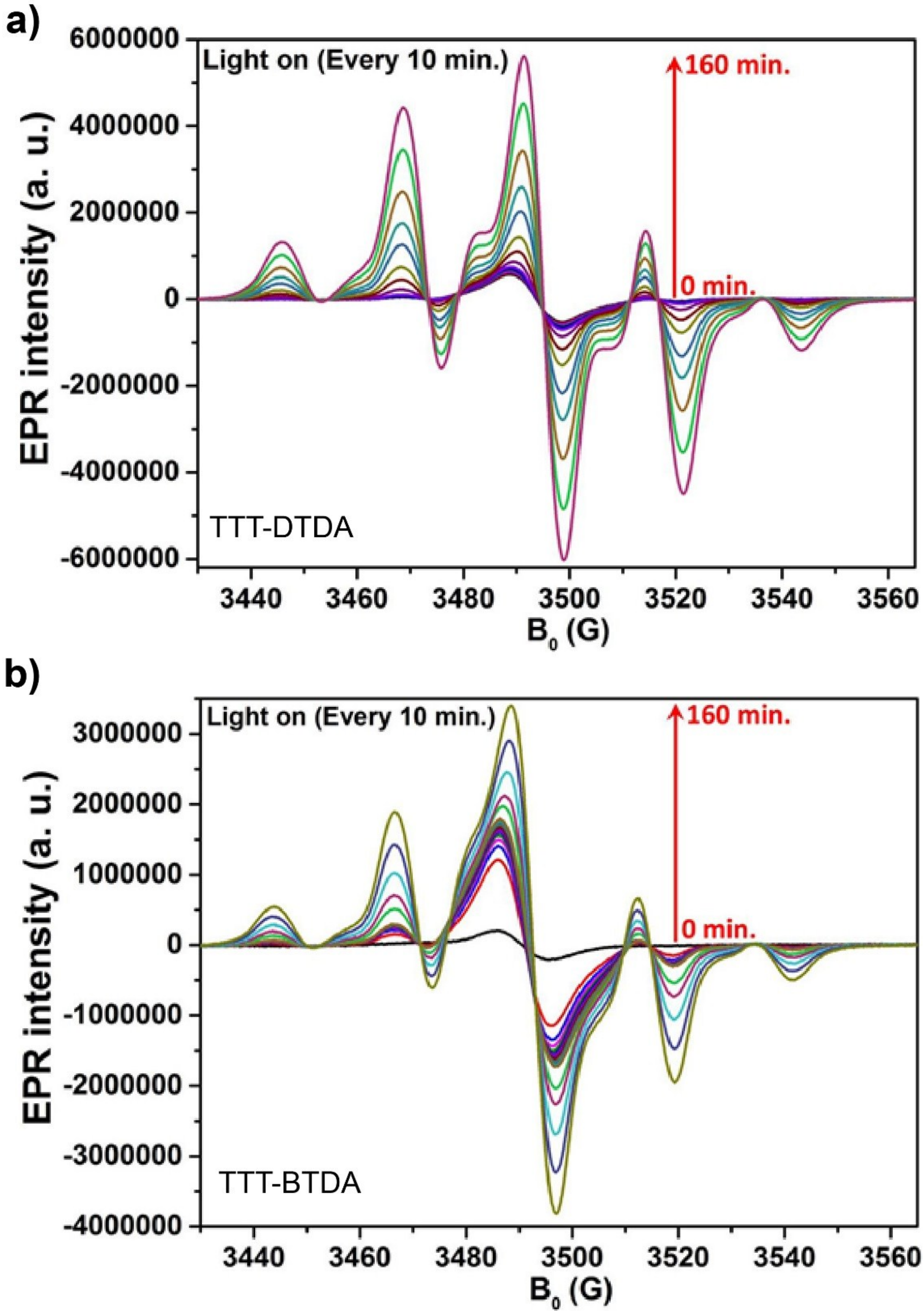

Figure S8. *In-situ EPR spectra of (a) TTT-DTDA and (b) TTT-BTDA measured every 10 minutes during UV-vis light irradiation. Reaction conditions: 5 mg COFs catalyst, 0.25 mL methyl methacrylate (MMA), 12.5 µL triethylamine (TEA) and UV-vis light irradiation using 300 W Xe lamp with an output power 1.5 W.*

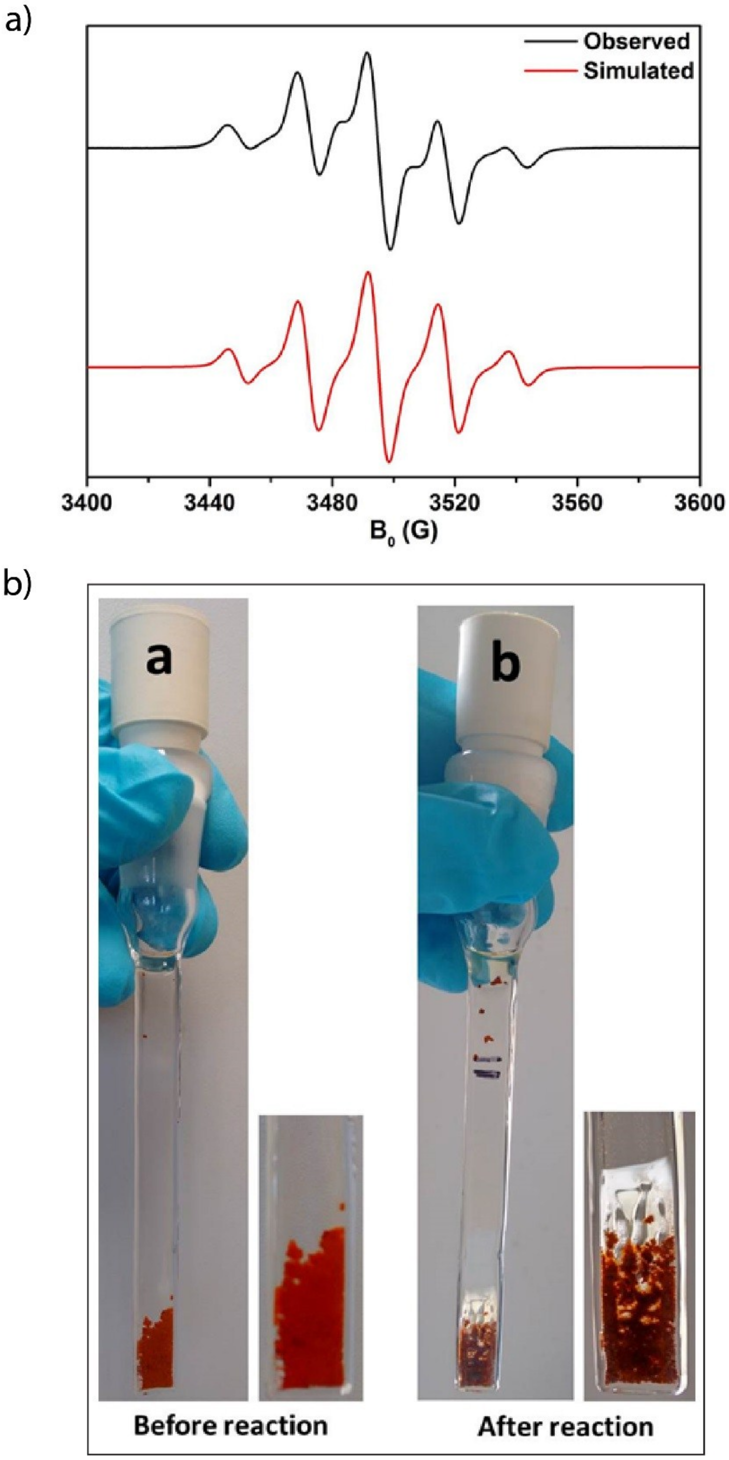

Figure S9. *(a) Experimental in situ EPR spectrum of TTT-DTDA measured after 160 min under photocatalytic conditions (cf. Figure S8a) and calculated spectrum using the following parameters: g = 2.0048, A_{CH3} = 22.2 G, $A_{H\beta 1}$ = 14.7 G, $A_{H\beta 2}$ = 7.9, line width 7.5 G. (b) Sample TTT-DTDA in the EPR flat cell before and after photocatalytic reaction for 160 min.*

Section S6. COF stability after radical polymerization of MMA to PMMA:

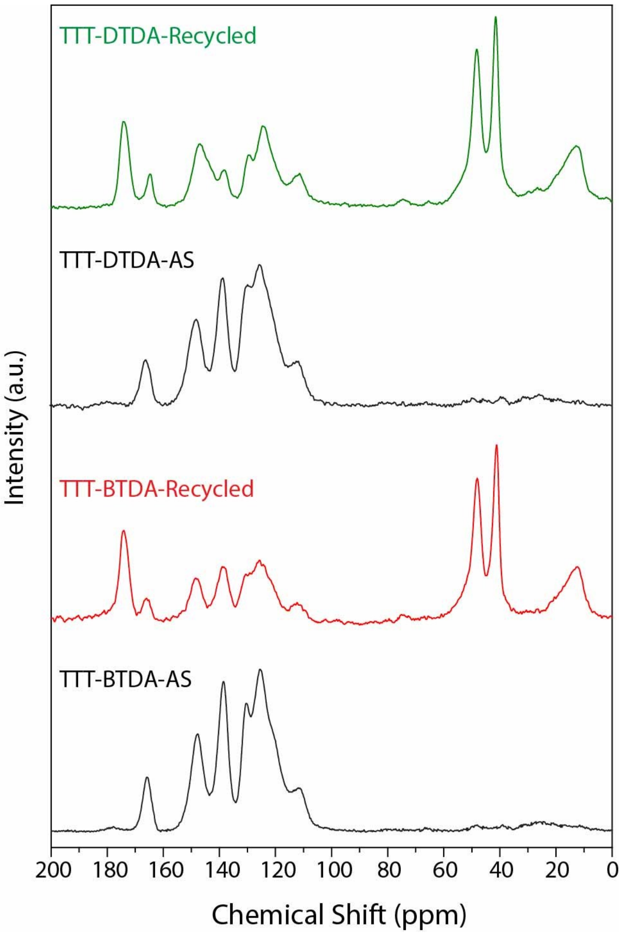

Figure S10. *^{13}C Solid-state cross-polarization magic angle spinning nuclear magnetic resonance (CP/MAS-NMR) spectroscopy analyses for TTT-DTDA and TTT-BTDA recycled after 2nd cycle of MMA polymerization compared with pristine samples, showing the complete preservation of the COF structural integrities and intact conjugated moieties within the COF backbone after photopolymerization. The peaks in aliphatic region at ~48, ~41 and ~12 ppm could be identified as signature peaks for unwashed PMMA from COF pore and bulk material.[5] The additional peaks (~175 ppm) of the recycled TTT-DTDA and TTT-BTDA COFs could be assigned to the carbonyl carbon peaks from the PMMA that has been immobilized in the COF matrix.*

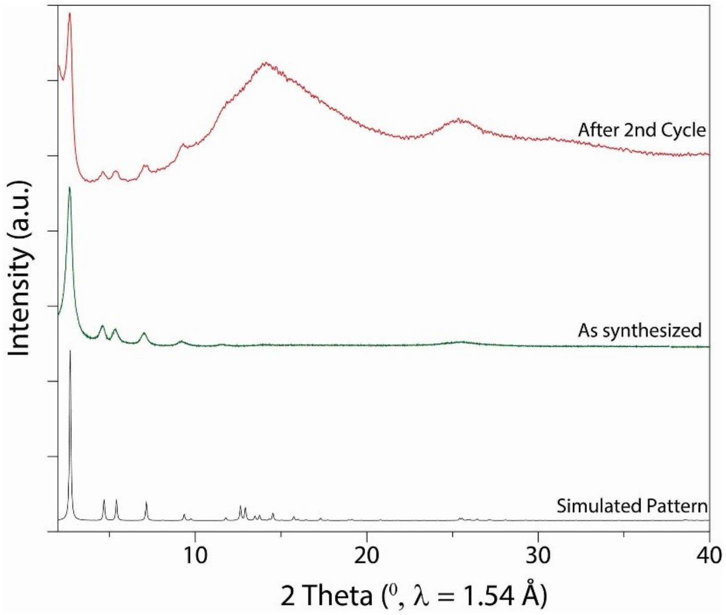

Figure S11. *Powder X-ray diffraction patterns for TTT-DTDA initiator as compared with as synthesized and simulated patterns confirming the significant preservation of the COF structure after 2 cycles of radical polymerization. The broad peak between 10-20° (2θ) can be assigned to PMMA polymer preset in COF matrix.*

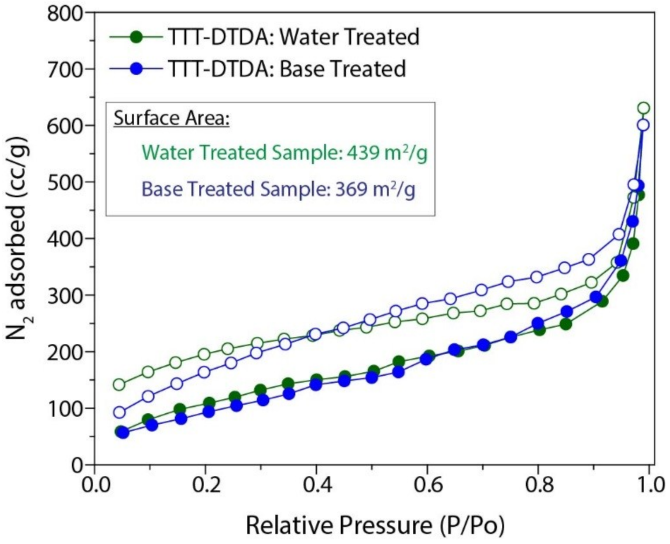

Figure S12. *Nitrogen adsorption isotherms of water and NaOH (1 N) treated TTT-DTDA COF samples*

Section S7. Structure modelling and atomic coordinates of COFs:

The structure models were generated using the *Material Studio Modelling* 5.0 package.[6] The geometry was optimized using the Forcite module and UFF forcefield.[7] Full profile pattern fitting (Pawley) was performed against the experimental powder pattern using the *Reflex* module.

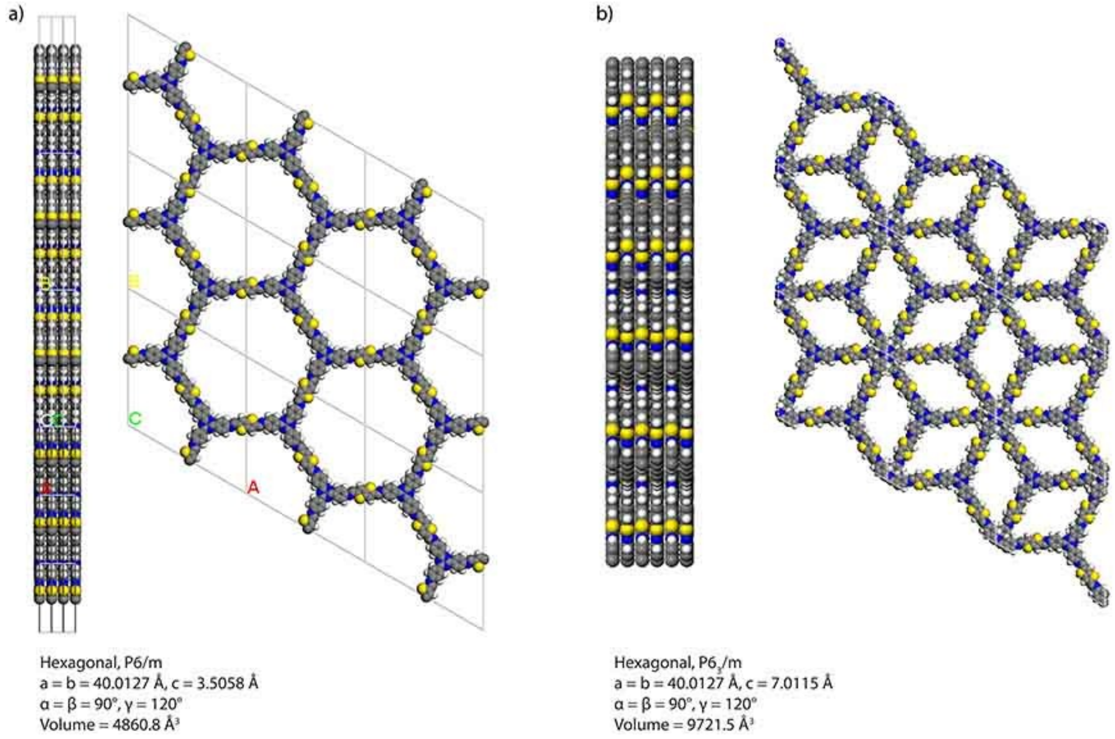

Figure S13. *The computationally determined structures of TTT-DTDA. a) The theoretical and side view of TTT-DTDA with eclipsed (AA) stacking arrangement. b) The theoretical structure and side view of TTT-DTDA with staggered (AB) stacking arrangement.*

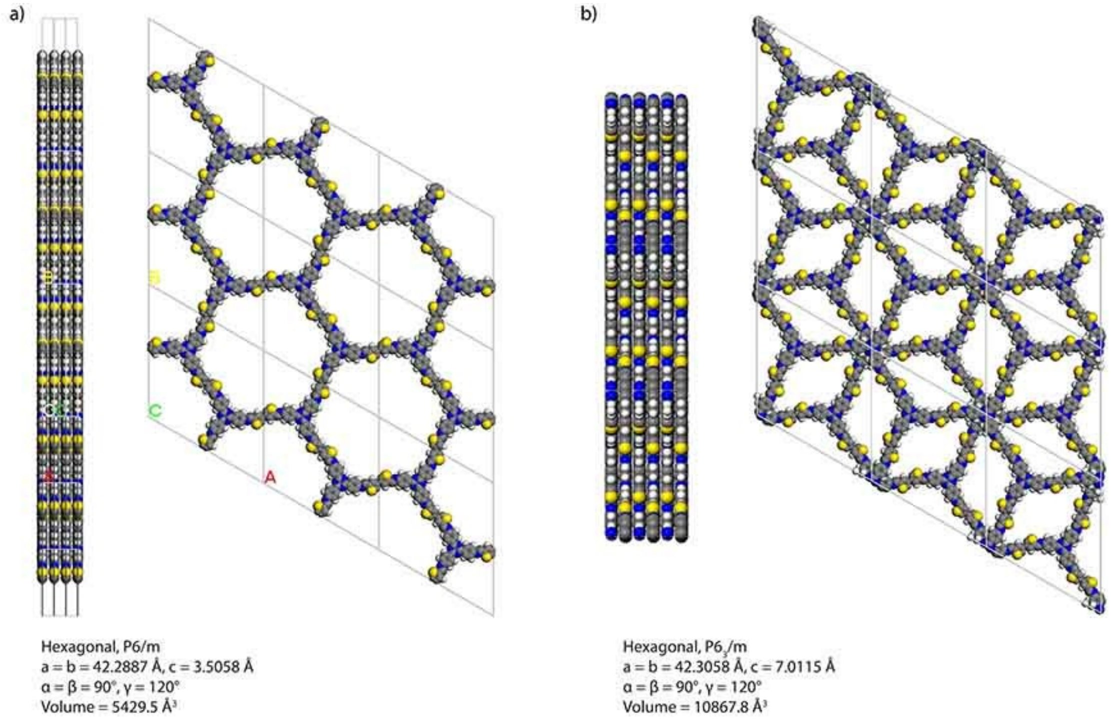

Figure S14. *The computationally determined structures of TTT-BTDA. a) The theoretical and side view of TTT-BTDA with eclipsed (AA) stacking arrangement. b) The theoretical structure and side view of TTT-BTDA with staggered (AB) stacking arrangement.*

In order to reveal the structures of these COFs and to calculate the unit cell parameters, possible 2-dimensional (2D) models were optimized using Density Functional Tight-Binding method (Figure S5 and S6). Several stacking possibilities were considered for calculating the model structures.[8] We found that, the experimental PXRD patterns are nicely matching with the simulated patterns of some near-eclipsed (AA) stacking models (Figure S11). Due to aforementioned reasons and comparing the experimental and simulated PXRD patterns, we propose the structures of TTT-DTDA and TTT-BTDA close to hexagonal space group (*P*6/*m*). Refinements of PXRD pattern were done using Reflex module of Material studio.[6]

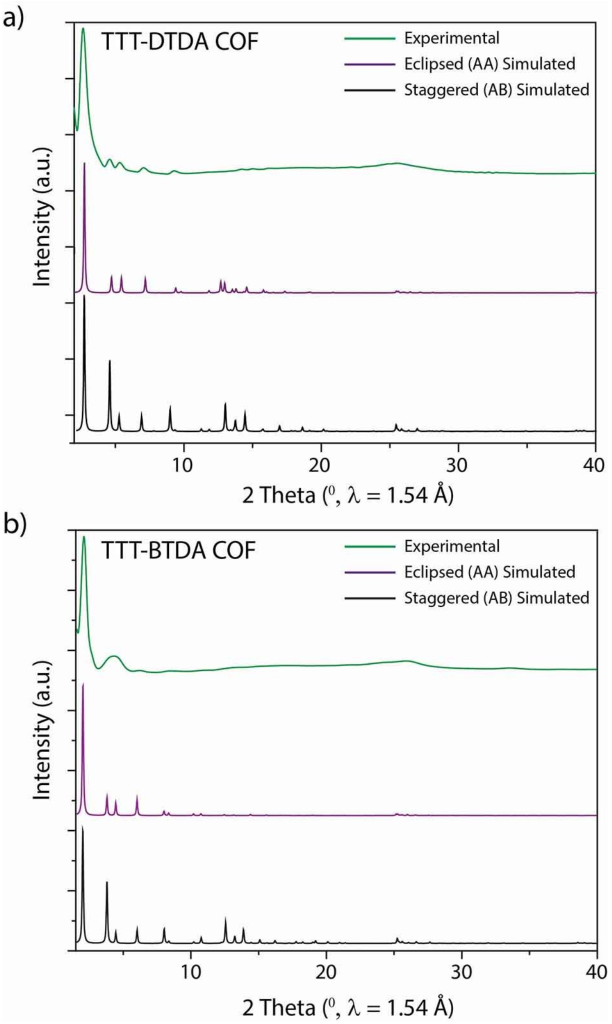

Figure S15. *The comparison between simulated and experimental PXRD patterns for: a) TTT-DTDA, and b) TTT-BTDA COFs, showing the good match between the experimental PXRD data with the eclipsed (AA) stacking model.*

Table S4. *Fractional atomic coordinates for TTT-DTDA COF:*

**TTT-DTDA COF**<br>Space group symmetry: *P*6/m (175)<br>*a* = *b* = 40.0127 Å; *c* = 3.5058 Å<br>*α* = *β* = 90˚; γ = 120˚				
**Atom Name**	**Atom**	***x***	***y***	***z***
H1	H	0.53366	0.57786	1
N2	N	0.62775	0.31658	0
C3	C	0.74851	0.36856	0
C4	C	0.64433	0.29411	0
C5	C	0.65414	0.42044	0
C6	C	0.63681	0.44329	0
H7	H	0.68534	0.43455	0
H8	H	0.65476	0.47442	0
C9	C	0.40898	0.63709	0
C10	C	0.42626	0.61418	0
H11	H	0.42735	0.66816	0
H12	H	0.45744	0.62815	0
C13	C	0.59662	0.42631	0
S14	S	0.44219	0.48532	0
C15	C	0.45669	0.56174	0
C16	C	0.48907	0.45206	0
C17	C	0.48346	0.483	0
C18	C	0.47164	0.53501	0
N19	N	0.58008	0.4509	0

Table S5. *Fractional atomic coordinates for TTT-BTDA COF:*

**TTT-BTDA COF**<br>Space group symmetry: *P*6/m (175)<br>*a* = *b* = 42.2887 Å; *c* = 3.5058 Å<br>*α* = *β* = 90˚; γ = 120˚				
**Atom Name**	**Atom**	***x***	***y***	***z***
C1	C	0.51751	0.50013	1
C2	C	0.55122	0.53161	1
H3	H	0.50231	0.55838	1
N4	N	0.63027	0.32063	0
C5	C	0.7432	0.36005	0
C6	C	0.64279	0.29666	0
C7	C	0.66427	0.42105	0
C8	C	0.65111	0.44548	0
H9	H	0.69351	0.43192	0
H10	H	0.67026	0.47456	0
C11	C	0.39782	0.62969	0
C12	C	0.41092	0.60522	0
H13	H	0.41733	0.65868	0
H14	H	0.44013	0.61594	0
C15	C	0.61356	0.43266	0
S16	S	0.4128	0.47622	0
C17	C	0.43279	0.55011	0
C18	C	0.52058	0.46927	0
C19	C	0.44347	0.5221	0
N20	N	0.60128	0.45874	0

Section S8. Synthesis and Characterizations of IISERP-COF4:

The synthesis of IISERP-COF4 was performed by following the reported procedure.[3]

Typically, 17 mg of terephthalaldehyde (0.127mmol) was dissolved in 3 mL ethanol in a Pyrex tube. To this solution, 3.0 mL of O-dicholorobenzene was added and sonicated for 5 minutes. Then, 30 mg of 2,4,6-Tris(4-aminophenyl)-*s*-triazine (0.085mmol) and 0.25 mL of aqueous acetic acid (3 M) were added to the reaction mixture and contents were further sonicated for 5 minutes. The contents was flash frozen in a liquid nitrogen bath for 3 times and sealed under vacuum. The tube was placed in an oven and heated to 120 ºC for 3 days. The obtained yellow powder was washed with methanol and acetone, and finally dried at atmospheric conditions.

Yield: 50%

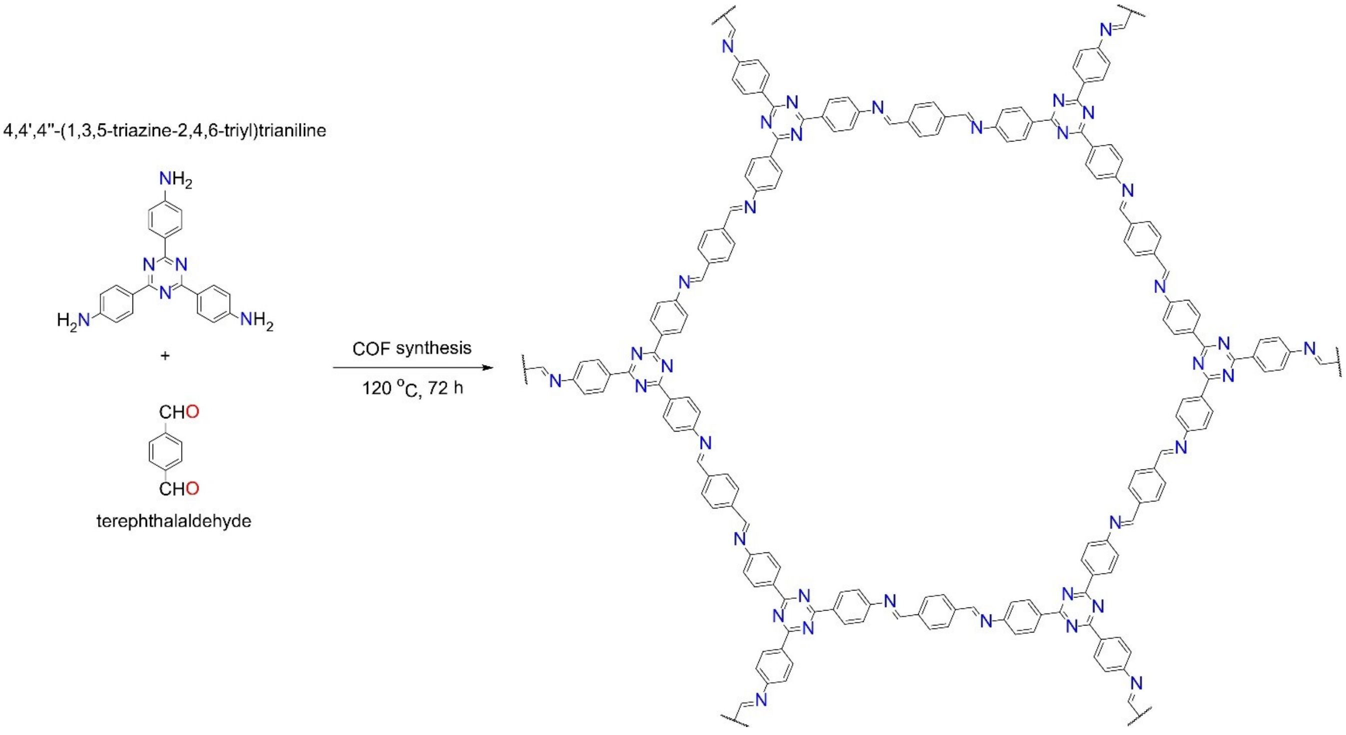

Figure S16. *Synthesis of IISERP-COF4 by following solvothermal method. The reaction of 4,4',4''-(1,3,5-triazine-2,4,6-triyl)trianiline and terephthaldehyde linker yielded IISERP-COF4, in presence of acetic acid as catalyst.*

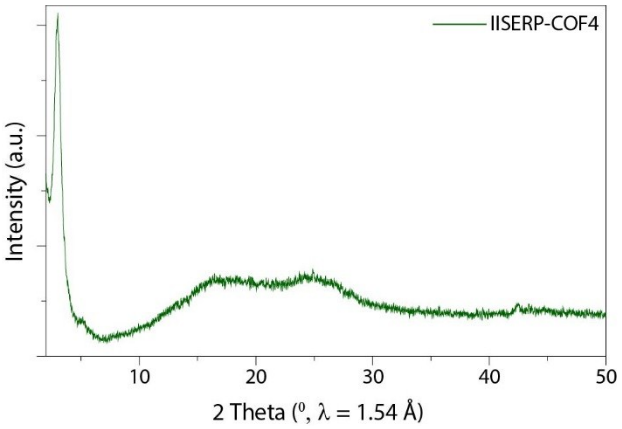

Figure S17. *Powder X-ray diffraction pattern of IISERP-COF4 showing similar pattern to those reported in literature.*

Table S6. *Comparison of radical polymerization performance for reported heterogeneous photoinitiators:*

Serial Number	Name of Material	Conversion (%)	Time (h)	Reference
1	RAFT: MTTCD	59.5	10	*J. Memb. Separ. Tech*. **2012**, *1*, 117-128.
2[(a)]	GP-5	64	6	*Nanoscale Res Lett.* **2014**, *9*, 345.
3	ACP-MCM-41	49.4	3	*Polymer Journal*, **2009**, *41*, 672–678.
4	MMA:TPPT:AIBME = 100:2:1	42	12	*Polymers* **2017**, *9*, 608
5	CuBr/HMTETA (1:2)	66	6.25	*Ind. Eng. Chem. Res.* **2005**, *444*, 677-685.
6	Poly(S-benzoyl O-ethylmethacrylate xanthate)	46.5	0.66	*Eur. Polym. J.* **1993**, *29*, 63-68.
7[(b)]	P-25 TiO_2	52	18	*J. Am. Chem. Soc.* **2016**, *138*, 4330−4333.
8[(b)]	UiO-66-NH_2	13	18	*J. Am. Chem. Soc.* **2016**, *138*, 4330−4333.
9[(b)]	MOF-901	87	18	*J. Am. Chem. Soc.* **2016**, *138*, 4330−4333.
10	TX-CMP	19	4	*Macromolecules* **2014**, *47*, 4607−4614.
11	TX-TPM polymer	31	4	*Macromolecules* **2014**, *47*, 4607−4614.
12	TX-Ph polymer	25	4	*Macromolecules* **2014**, *47*, 4607−4614.
13	*g*-C_3N4	~30	6	*ACS Macro Lett.* **2012**, *1*, 546−549.
14	*IISERP-COF4*	07	12	This Work
14	TTT-DTDA	63	12	This Work
15	TTT-BTDA	54	12	This Work

(a) Polymerization of MMA using TPEBMP at 80°C in DMF using DGO-Br.

(b) The radical polymerization activity was carried out in presence of ethyl α-bromophenylacetate.

Section S8. References

1 S. Dadashi-Silab, H. Bildirir, R. Dawson, A. Thomas and Y. Yagci, *Macromolecules*, 2014, **47**, 4607–4614.

2 B. Kiskan, J. Zhang, X. Wang, M. Antonietti and Y. Yagci, *ACS Macro Lett.*, 2012, **1**, 546–549.

3 D. Mullangi, S. Shalini, S. Nandi, B. Choksi and R. Vaidhyanathan, *J. Mater. Chem. A*, 2017, **5**, 8376–8384.

4 S.-Y. Ding, J. Gao, Q. Wang, Y. Zhang, W.-G. Song, C.-Y. Su and W. Wang, *J. Am. Chem. Soc.*, 2011, **133**, 19816–19822.

5 E. F. Mccord, W. L. Anton, L. Wilczek, S. D. Ittel, L. T. J. Nelson, K. D. Raffell, J. E. Hansen and C. Berge, *Macromol. Symp.*, 1994, **86**, 47–64.

6 A. S. Accelrys, Material Studio Release Notes, Release 5.0, 2008.

7 A. K. Rappe, C. J. Casewit, K. S. Colwell, W. A. Goddard and W. M. Skiff, *J. Am. Chem. Soc.*, 1992, **114**, 10024–10035.

8 B. Lukose, A. Kuc and T. Heine, *Chem. - A Eur. J.*, 2011, **17**, 2388–2392.

Author Contributions

All authors contributed extensively to the work presented in this paper. P.P. and A.T. conceived the research project. P.P. conducted the synthesis of COFs and performed the characterizations. P.P. and A.A. designed the photocatalysis experiments and evaluated the radical polymerization of methyl methacrylate (MMA) to poly-methyl methacrylate (PMMA). J.S. administrated the project and performed and evaluated the XPS analyses for all the samples. M.-Y.Y. performed the SEM analyses for COF samples. J.R. performed the simulations, designed the ideal COF structures and evaluated the PXRD patterns. R.P.S. and A.B. performed the EPR analyses. The whole project was administrated by A.T., with the help from P.P. The manuscript was written by P.P. and A.T. with the input from the other authors.
